# A Comparison of Mainstream Genotyping Platforms for the Evaluation and Use of Barley Genetic Resources

**DOI:** 10.3389/fpls.2019.00544

**Published:** 2019-04-26

**Authors:** Benoit Darrier, Joanne Russell, Sara G. Milner, Pete E. Hedley, Paul D. Shaw, Malcolm Macaulay, Luke D. Ramsay, Claire Halpin, Martin Mascher, Delphine L. Fleury, Peter Langridge, Nils Stein, Robbie Waugh

**Affiliations:** ^1^School of Agriculture and Wine, The University of Adelaide, Adelaide, SA, Australia; ^2^School of Life Sciences, University of Dundee, Dundee, United Kingdom; ^3^Cell and Molecular Sciences, The James Hutton Institute, Dundee, United Kingdom; ^4^Leibniz Institute of Plant Genetics and Crop Plant Research, Gatersleben, Germany; ^5^Information and Computational Sciences, The James Hutton Institute, Dundee, United Kingdom; ^6^Center of Integrated Breeding Research, Georg-August University, Göttingen, Germany

**Keywords:** germplasm evaluation, GBS, SNP-array, diversity, GWAS

## Abstract

We compared the performance of two commonly used genotyping platforms, genotyping-by-sequencing (GBS) and single nucleotide polymorphism-arrays (SNP), to investigate the extent and pattern of genetic variation within a collection of 1,000 diverse barley genotypes selected from the German Federal *ex situ* GenBank hosted at IPK Gatersleben. Each platform revealed equivalent numbers of robust bi-allelic SNPs (39,733 and 37,930 SNPs for the 50K SNP-array and GBS datasets respectively). A small overlap of 464 SNPs was common to both platforms, indicating that the methodologies we used selectively access informative polymorphism in different portions of the barley genome. Approximately half of the GBS dataset was comprised of SNPs with minor allele frequencies (MAFs) below 1%, illustrating the power of GBS to detect rare alleles in diverse germplasm collections. While desired for certain applications, the highly robust calling of alleles at the same SNPs across multiple populations is an advantage of the SNP-array, allowing direct comparisons of data from related or unrelated studies. Overall MAFs and diversity statistics (π) were higher for the SNP-array data, potentially reflecting the conscious removal of markers with a low MAF in the ascertainment population. A comparison of similarity matrices revealed a positive correlation between both approaches, supporting the validity of using either for entire GenBank characterization. To explore the potential of each dataset for focused genetic analyses we explored the outcomes of their use in genome-wide association scans for row type, growth habit and non-adhering hull, and discriminant analysis of principal components for the drivers of sub-population differentiation. Interpretation of the results from both types of analysis yielded broadly similar conclusions indicating that choice of platform used for such analyses should be determined by the research question being asked, group preferences and their capabilities to extract and interpret the different types of output data easily and quickly. Access to the requisite infrastructure for running, processing, analyzing, querying, storing, and displaying either datatype is an additional consideration. Our investigations reveal that for barley the cost per genotyping assay is less for SNP-arrays than GBS, which translates to a cost per informative datapoint being significantly lower for the SNP-array.

## Introduction

The detection of genome-wide sequence-defined single nucleotide polymorphism-arrays (SNPs) is key to addressing a wide range of biological and ecological questions, from describing and partitioning overall levels of biological diversity to cloning genes conferring phenotypic traits, and for practical exploitation in animal and crop breeding. Alternative SNP alleles can be detected using a range of technologies that can be broadly classified into two types, semi-open and closed, based on the nature of the data generated. Closed systems can be represented by hybridization-based commercial SNP-arrays (Bayer et al., 2017) where the same panel of SNPs is repeatedly assayed for variation across all experiments and all germplasm. Semi-open systems are typified by RAD-seq (Miller et al., 2007), DArTseq (Kilian et al., 2012) or genotyping-by-sequencing (GBS, Elshire et al., 2011). These are similar in nature and assay new variation in each different set of genetic material analyzed. They have been widely adopted by plant genetics and ecological communities largely due to their generic nature and low establishment costs. We use the term semi-open because all typically involve a genome complexity reduction step, in GBS for example through use of methylation sensitive restriction enzymes to selectively avoid highly repetitive genomic regions (thus introducing bias), and then short read next generation sequencing of the regions adjacent to the cleaved restriction sites. Their increasing popularity stems from them being species and sequencing platform agnostic, as well as being considered fast, cheap and informative (Lu et al., 2015). However, they are notorious for generating noisy data. At lower sequence depth, GBS data includes a large fraction of missing data which requires imputation and sometimes complex computational interpretation prior to subsequent analysis.

Our primary interests focus on the biology of barley (*Hordeum vulgare* ssp. *vulgare* and ssp. s*pontaneum*), a large genome (∼5 Gbp) inbreeding diploid (2n = 2x = 14) species that was one of the worlds’ first domesticated crop plants (Snape et al., 2013). Along with large sections of the barley genetics community, for the last 15 years we have generally favored the application of robust SNP-based assay platforms (in our case developed by Illumina) for high throughput genotyping requirements (Close et al., 2009; Comadran et al., 2012). The most recent iteration of this platform is a 50K iSelect custom genotyping array (Bayer et al., 2017) which includes SNPs selected from a combination of previously informative ‘legacy’ Illumina platform SNPs (for backward compatibility), supplemented with a large cohort derived from comprehensive exome capture sequence data (Russell et al., 2016). They were chosen to represent the range of diversity observed in domesticated barley (landraces and cultivars) from across the geographic range of the species, to have minor allele frequencies (MAFs) of > 5% in the ascertainment population, be located in annotated, physically and genetically mapped positions and to be well-distributed across each of the seven barley chromosomes. We chose the Illumina iSelect SNP platform as it has proven to be a highly robust technology that yields exceptional data quality with few missing values^1^. For many applications this is crucially important because all new data are backward compatible, easily extracted, quality checked, databased and referenced over time, with the computational load between data generation and use for analysis being minimal and therefore suited to almost anyone in a research or breeding environment. Data quality control (QC) and error checking is consistent and straightforward. However, SNP-arrays suffer from ascertainment issues (Moragues et al., 2010), are species specific and therefore need to be developed independently for each crop. Their development and testing can be lengthy requiring considerable prior knowledge and adding new SNPs to the platform is difficult and expensive. Even after development, the service cost of a single genotyping assay is widely considered to exceed that of GBS.

Given that both approaches appear to have certain advantages we were keen to critically explore how they each performed in a suitably sized comparative experiment focused on the evaluation of a diverse barley germplasm collection. We also wanted to explore their affinity for subsequent applications of the derived data, such as candidate gene identification using population-based approaches. We consider these questions important not only in our own research programs and in those of the barley genetics community, but for comparative purposes we were keen to carefully establish the level of concordance between different data types. This is an important and fundamental question given that large germplasm characterization projects are currently being undertaken by the plant genetics community (e.g., Lu et al., 2015; Yu et al., 2016^2^) and these are dominated by the use of semi-open systems, mainly GBS. We wanted to understand and clarify the advantages and caveats associated with each platform and the resulting data types and ask whether the overall values afforded by one approach outweighed those of the other. We were motivated to do this by the recent genotypic characterization of the entire 22,000 genotypes in the IPK Gatersleben barley GenBank collection (Milner et al., 2019) that was achieved using the widely adopted *Pst*I-*Msp*I GBS protocol (Wendler et al., 2014). The use of GBS for ‘germplasm genomics’ could be considered a surrogate for whole genome (shotgun) sequencing (WGS), which at the moment at least, is not considered financially feasible in large genome crops like maize (∼2.5 Gbp), barley (∼5 Gbp), and wheat (∼17 Gbp), in contrast to rice (∼370 Mbp, Wang et al., 2018) and Arabidopsis (∼135 Mbp, The 1001 Genomes Consortium, 2016). This is because the latter genomes are suitably compact for WGS to be affordable, and for rice in particular, the importance of the crop sufficiently great to justify the ‘gold standard’ investment.

When GBS was becoming popular in the plant community, we evaluated the *Pst*I-*Mse*I GBS protocol in a study aimed at mapping the barley *breviaristatum-e* (dwarfing) locus in a bi-parental recombinant inbred line (RIL) population (Liu et al., 2014). In that work, after highly conservative and rigorous filtering to remove missing data, low MAF, dominant/null and heterozygous SNPs, we ended up with a total of 1,949 robust and co-dominant markers that could be confidently used for genetic analysis. Analysis of the same population with the recent 50K SNP-array revealed 14,626 robust SNP allele calls (Bayer et al., 2017), on the face of it a significant improvement on marker number, which subsequently provided improved genetic resolution. While possible explanations for this could be our conscious bias in SNP ascertainment when developing the 50K array, or reflect the quality and depth of our GBS data, we considered the extent of this numerical discrepancy further motivation for the current investigation.

As a result, the primary questions we address here are: how do these platforms, generic GBS and a species-specific SNP-array, perform in relative terms in barley, how concordant are the outputs, how easy are they to compare between datasets and labs, are there clear cases where one approach provides significant advantages over the other, and what are realistic cost comparators? We believe that the answers to these and other questions will be important for both ourselves and others in the barley community who may tend to favor one technical approach over another. It is also a critical evaluation of how different data types are being assessed and interpreted in similar, ongoing studies throughout the plant community. We used the same *Pst*I-*Msp*I GBS dataset described in Milner et al. (2019) and the representative diversity sub-set of 1,000 of the >22,000 barley genotypes described in their recent IPK GenBank study. We genotyped these 1000 lines with the barley 50K iSelect array and used the resulting datasets as the basis for our evaluations.

## Materials and Methods

### Genetic Materials: 1,000 Genotype Set

Selection of a core set of 1,000 genotypes from an extended collection of 22,626 genotypes lodged in the German Federal *ex situ* GenBank hosted at IPK Gatersleben was done exactly as described by Milner et al. (2019), based on GBS data with CoreHunter3^3^ and the average entry-to-nearest-entry criterion. It was done after analysis of population structure and genetic similarity, and imputation of missing values in the GBS genotype matrix using FILLIN with default parameters (Swarts et al., 2014; Milner et al., 2019).

### SNP Genotyping and Analysis

We used exactly the same GBS dataset described by Milner et al. (2019) and exactly the same DNAs as used in that study were submitted for SNP genotyping using the barley Illumina 50K iSelect SNP platform (Bayer et al., 2017). The GBS SNP matrix of Milner et al. (2019) is accessible from https://doi.ipk-gatersleben.de/DOI/ecfbdb3d-4882-406c-9e82-7758ed5395c7/4f58176f-4824-4c32-bca1-3d87500d82f3/2. The filter criteria for inclusion of GBS SNPs used by Milner et al. (2019) were: (i) up to 10% missing genotype calls; (ii) up to 10% heterozygous calls; and (iii) the number of heterozygous calls does not exceed the number homozygous minor allele counts.

For the 50K data, prior to sample submission, DNA quality was assessed using a Nanodrop 2000 (Thermo Fisher Scientific, Waltham, MA, United States) with a requirement for 260/280 and 260/230 ratios to be > 1.8. DNA was then quantified using Picogreen (Thermo Fisher Scientific, Waltham, MA, United States) and 300 ng DNA per sample lyophilized and sent to Geneseek (Neogen Europe, Ltd., Auchincruive, United Kingdom) for Illumina HTS processing and HiScan array imaging (Illumina, San Diego, CA, United States). SNP alleles were called using GenomeStudio Genotyping Module v2.0.2 (Illumina, San Diego, CA, United States) and the resulting data investigated and analyzed as previously reported for 9K Illumina data (Milne et al., 2010; Comadran et al., 2012). The physical positions of both GBS and 50K SNPs were assigned by BLAST (Altschul et al., 1990) according to their position on the 2017 barley genome assembly (Mascher et al., 2017).

### Genetic Analyses

Both the GBS and SNP-array genotyping matrices were formatted for analysis using PLINK1.9^3^ (Chang et al., 2015). Flags: –maf was used to compute minor allele frequency value and extract selected SNPs; –geno was used to extract SNPs with less than the desired percentage of missing values; –pca was used to compute principal components analysis (PCA) with the var-wts option to extract variant weight; and the –recode flag to create the VCF file to compute π in Vcftools (Danecek et al., 2011). Sliding window analyses were performed using an in-house Perl script ‘plotDensity,’ obtained from F. Choulet (INRA Clermont Ferrand) with the following parameters: a window of 10 Mbp and a step of 1 Mbp. D′ and R2 were computed with snpStats R package (Clayton, 2017) and all figures were generated using the R environment with ggplot^3^ packages (Wickham, 2016). Genome-wide association scan (GWAS) analysis was conducted using the compressed mixed linear model (Zhang et al., 2010) implemented in the GAPIT R package (Lipka et al., 2012; Tang et al., 2018). The phenotypic data were also the same as used in Milner et al. (2019) except for the categorical scores for growth habit (winter vs. spring) which were prior genotype classifications. The FDR-corrected *p*-values were used to draw Manhattan plots using qqman R package (Turner, 2014). We used the first three principal components (PC’s) in the GWAS model for population correction. A Mantel test for correlation between distance matrices computed from GBS and 50K data was performed using mantel.rtest function of the R package ade4. Allele counts and frequencies were verified using in-house python script.

Discriminant analysis of principal component (DAPC) was conducted as described in Jombart and Collins (2015). First, clusters were identified using find.clusters function. According of the results of the PCA, *k* = 3 (GP1, GP2, and GP3) was chosen, and 400 axes (n.pca, accounting for > 80% of the cumulative variance) retained for the discriminant analysis. The latter was run using the function dapc with all of the retained eigenvalues. Cluster assignation was used to create subpopulations. Fst estimates between subpopulations were calculated according to Weir and Cockerham (1984). Regions of genetic differentiation between subpopulations was displayed as Manhattan plots based on −1/log_10_Fst values of individual markers plotted linearly along each chromosome according to physical position.

## Results

### SNP Metrics

Among the 43,461 scorable assays on the 50K SNP-array in this experiment we extracted 42,300 (96%) robust and polymorphic SNPs after examination of genotype clusters in GenomeStudio. For comparison, the GBS genotype matrix was comprised of the 37,327 high quality polymorphic bi-allelic GBS SNPs described in Milner et al. (2019). We removed 2,567 SNP in the 50K SNP-array dataset and 603 SNP in the GBS dataset for all subsequent comparative analysis due to their not being assigned a physical position on the current barley genome pseudomolecules (Mascher et al., 2017). The final datasets for comparison therefore comprised 39,733 and 37,327 SNP distributed on the seven chromosomes of the barley genome for 50K SNP-array and GBS datasets respectively. Only 464 markers were in common in both datasets illustrating the fact that each assay platform accesses different regions of the barley genome. The distribution of SNPs among the seven chromosomes was only slightly different (Table 1) with more markers from the 50K SNP-array on chromosomes 2H (+685), 4H (+506), 5H (+1,702), and 6H (+313) and more markers from GBS on 1H (+345), 3H (+71), and 7H (+384). Overall SNP densities along each chromosome for both datasets were broadly similar, with only two differences apparent on the short arms of chromosomes 4H and 5H where the SNP density was higher on the 50K dataset (Supplementary Figure S1).

**TABLE 1:** Single nucleotide polymorphism-arrays (SNPs) marker distribution.

(A) Number of SNPs per chromosome according to assay platform.										

	1H	2H	3H	4H	5H	6H	7H	Anchored	Un	Total
50K	4,364	6,564	6,076	4,668	7,333	4,937	5,791	39,733	2,567	42,300
GBS	4,709	5,879	6,147	4,162	5,631	4,624	6,175	37,327	603	37,930

**(B) Distribution of monomorphic and polymorphic SNPs according to assay platform and germplasm classification.**										

			**50K**					**GBS**		
			**Landrace**		**Cultivar**			**Landrace**		**Cultivar**

Monomorphic			26		24			1,617		11,849
Polymorphic			39,707		39,709			35,710		25,478

Both datasets revealed a high number of markers in the distal regions of each chromosome decreasing markedly in the peri-centromeric/centromeric regions. As this could be biased due to the distribution of genes, we partitioned our analysis according to genic and intergenic markers. In accordance with the origin of the information for the 50K assay being from exome capture data we observed a large number of SNPs in genic regions, with 72% (28,875) associated with genes and present at higher density in the gene-rich distal regions of each chromosome (Supplementary Figure S1). In contrast, the GBS dataset showed a reduced representation with 52% (19,436) of SNPs in genic regions, generating more of a balance between genic and intergenic SNPs. With both platforms, as expected, gene associated markers remain more frequent in the distal parts of all chromosome arms, a feature that was especially apparent in the 50K dataset.

### Allele Frequencies and Diversity Statistics

**FIGURE 1:**
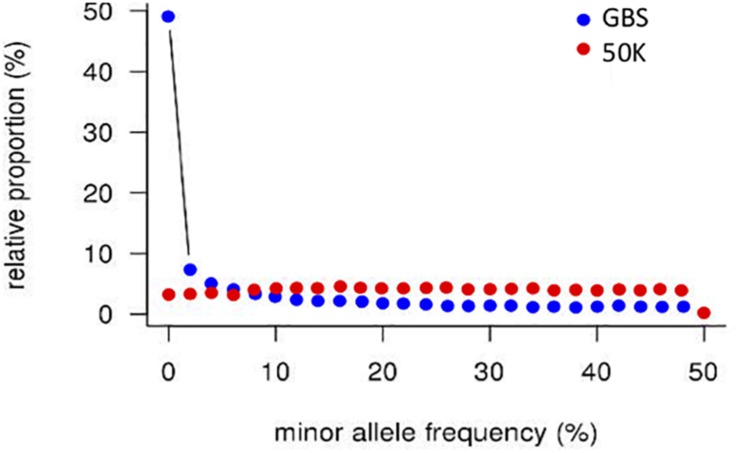
Distribution of minor allele frequencies in GBS and 50K array data. SNP counts were aggregated in 2% bins.

**FIGURE 2:**
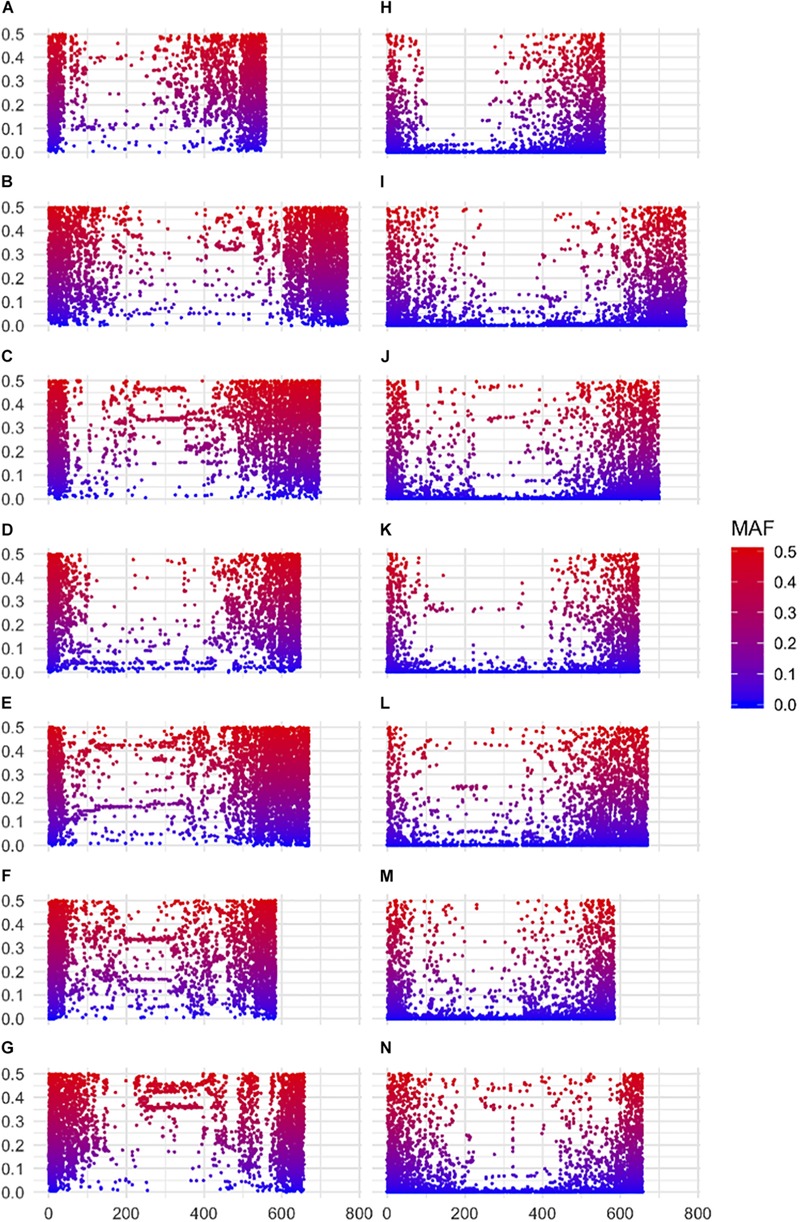
Minor allele frequencies according to physical location of markers along barley chromosomes. **(A–G)** 50K SNP-array data for chromosomes 1H–7H top to bottom respectively (left panel) and **(H–N)** GBS data chromosomes 1H–7H top to bottom respectively (right panel). SNPs are color coded according to MAF.

**FIGURE 3:**
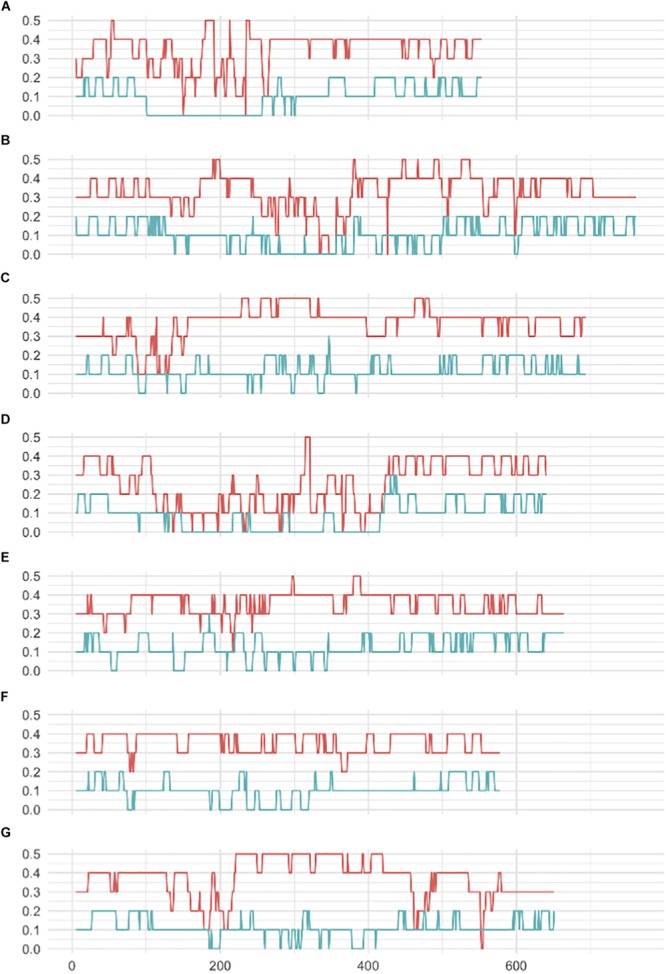
Sliding window analysis of genetic diversity (π) **(A–G)** seven barley chromosomes 1H–7H respectively. Red, 50K data; Blue, GBS data.

A striking difference between datasets was the high proportion of GBS markers associated with a low minor allele frequency (MAF) compared to the 50K dataset. The percentage of markers with a MAF < 1 or <5% was 1.2 and 7.6% for the 50K chip but reached 41.6 and 58.9% for GBS respectively (Figure 1). Across the genome, we did not observe specific regions on the physical map with low MAF (Figure 2). However, a pattern in the centromeric regions, visible as horizontal tracks in the pericentromeric regions in Figure 2, mainly in the 50K data, is likely the result of a limited number of extended conserved centromeric haplotypes within the current germplasm set (i.e., high linkage disequilibrium between sets of markers). Exploring MAF further, in the GBS dataset we found 11,849 SNP with a MAF of < 1% in the landraces and 1,617 in the cultivars, emphasizing the value of GBS for detecting low frequency variants in more diverse materials. In stark contrast, only 24 and 26 SNP exhibited a MAF of < 1% in landraces and cultivars, respectively, in the data from the 50K SNP-array, likely reflecting the 50K chip design strategy that prioritized markers with MAFs of > 5% in the SNP ascertainment (i.e., landrace and cultivar) genepool. Finally, we examined the degree of polymorphism along each chromosome within the population revealed by each marker type by calculating the nucleotide diversity statistic π (Nei and Li, 1979). Along all chromosomes the 50K SNP chip data consistently revealed higher levels of diversity than GBS (Figure 3), presumably reflecting the low MAF of the latter.

### Overall Patterns of Genetic Diversity

**FIGURE 4:**
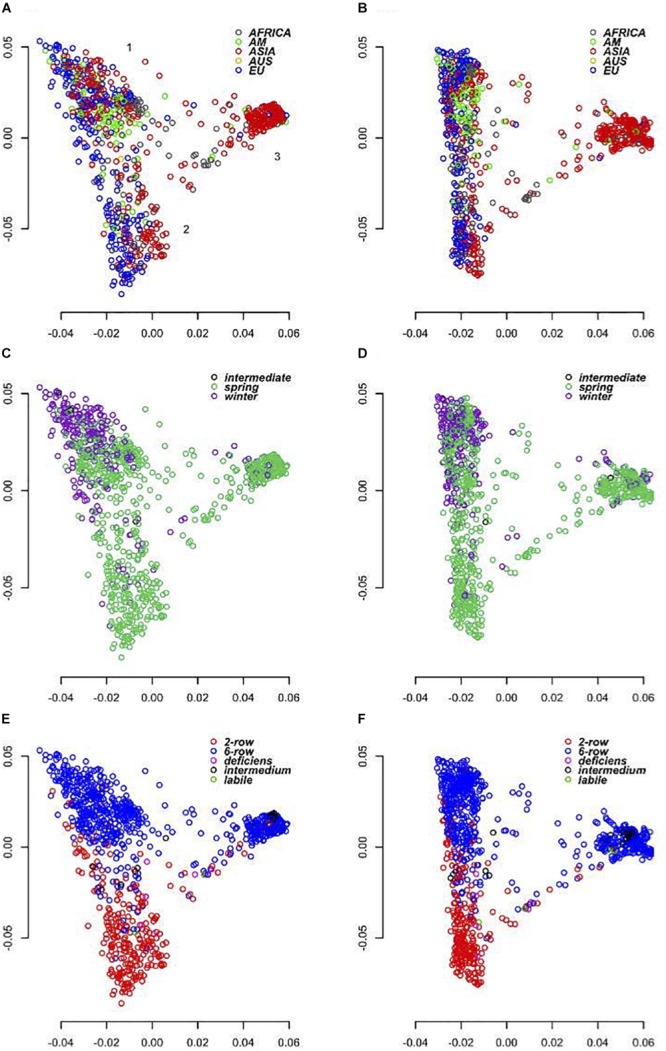
Principal coordinates analysis. **(A,C,E)** Shows diversity revealed by the 50K SNP-array and **(B,D,F)** by GBS. **(A,B)** Shows genotypes color coded according to geographical origin, **(C,D)** according to growth habit and **(E,F)** according to row-type.

As the SNPs on the 50K chip were largely ascertained from the European domesticated gene pool (cultivars and landraces), we expected that its use across a wider spectrum of material may reveal biases associated with the origin of the data used in its design. We were therefore keen to investigate the degree of concordance between data generated by the ‘closed’ 50K platform with that from the semi-open GBS approach. To assess how each platform performs in diversity analyses we ran PCAs using the 39,733 (50K) and 37,327 (GBS) SNP marker datasets (Figure 4). We initially compared three different analysis softwares, SNPRelate (Zheng et al., 2012), Eigensoft6 (Price et al., 2006), and PLINK1.9 (Chang et al., 2015) in each case partitioning the diversity into 10, 20, 32, and 50 PC’s. All gave virtually identical results with the percentage variation explained by the first PC’s decreasing as the number of PC’s increased. As the spatial distribution of the individuals over the first 3 PC’s was otherwise maintained in each treatment of the data, we report here only the results from using PLINK1.9 and the first 20 PC’s. In this analysis the first two PC’s explained together 44.3% of the variance for the 50K array and 33.71% of the variance for GBS (Figure 4). Both datasets show that PC1 broadly separates eastern and western barleys, defining two distinct genetic pools. PC2 was more, but not exclusively related to the differentiation between 2-row and 6-row inflorescence architectures and explained a lower level of variation in the GBS data (9.58%) compared to the 50K (19.59%). PC2 is also correlated with different growth habits in the western barley material with the vast majority of the six row-types having a winter growth habit. Overall, the results of PCA on GBS and 50K data were concordant, and the correlation of genetic distance matrices was high (*r* = 0.62, *p* = 0.0001; 99 permutations, Mantel test).

### Genome-Wide Association Scans

**FIGURE 5:**
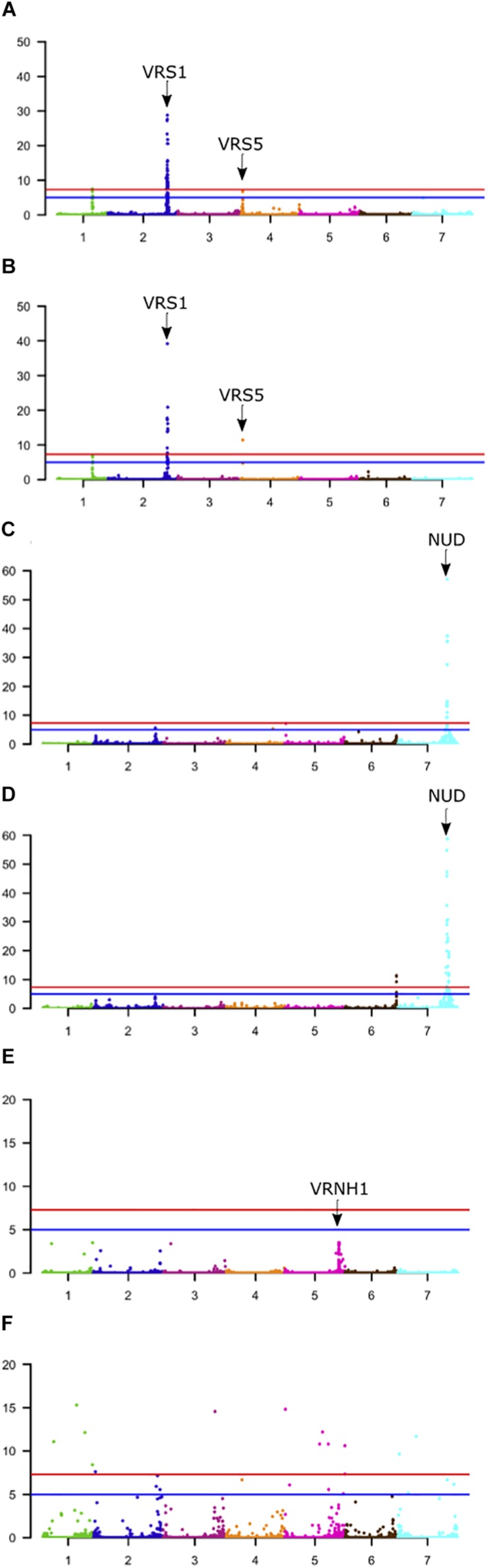
Genome-wide association scans (GWASs) of phenological traits segregating in the 1000 core population. **(A,C,E)** Use the 50K array data. **(B,D,F)** Use GBS data. **(A,B)** Data for row-type, **(C,D)** non-adhering hull, and **(E,F)** seasonal growth habit. Horizontal red line = –log10(5e-8), Horizontal Blue line = –log10(1e-5). The location of known genes associated with each trait is indicated. Y-axis; −log10(*p*) values.

One obvious application of high throughput genetic characterization of germplasm collections is to explore the possibility of using the data for trait related gene identification using population genetic approaches. Here, we chose GWAS initially to investigate two of the traits analyzed by Milner et al. (2019) (row-type, naked hull) along with binary scores for seasonal growth habit. In all cases we used legacy phenotypic data and compared the output from 50K and GBS datasets (Figure 5 and Supplementary Figure S2). Given the virtual exclusivity of the different marker types we wanted to establish if the same regions of the genome were identified using different markers and whether one marker type consistently provided higher resolution or greater support than the other. We chose these traits because at least some of the causal genes are well-established.

First, for row-type we detected three significant associations in both datasets (Figure 5A). Those on chromosome 2H were close (∼650 kbp away in each case) to the major row-type gene *SIX ROWED SPIKE1* (*HvVRS1*, Komatsuda et al., 2007) which is both necessary and sufficient to induce the two to six row inflorescence conversion in cultivated barley. Those on 4H were close to the modifier of lateral spikelet fertility gene *HvVRS5* (syn. *INTERMEDIUM-C*, Ramsay et al., 2011) which is epistatic to *HvVRS1*. In this case GBS outperformed the 50K chip in terms of resolution (closest marker 1.3 vs. 223 kbp from the target gene respectively). As in Milner et al. (2019) we also detected a highly significant association on chromosome 1H. This is distinct from the *HvVRS3* gene (Bull et al., 2017; van Esse et al., 2017) which also maps on chromosome 1H. It falls in a region with several strong inflorescence development candidate genes (Schnurbush et al., personal communication).

Second, in both wild barley and the majority of domesticated types, the awn-bearing lemma is firmly bound to the grain through a lipid-based cementing layer between the caryopsis and lemma (Taketa et al., 2008). Driven largely for use as food either during or soon after domestication, over time individuals were selected where the tough and fibrous hull separated easily from the harvested grain. These so-called naked barleys carry a loss-of-function allele in the *NUDUM* (*HvNUD*) gene, an ethylene response factor required to promote formation of the cementing layer. Using lemma adherence to the grain as a binary character we again performed a GWAS with each marker type. The most highly associated marker on the 50K SNP-array was some 44 kbp distal to *NUD* on chromosome 7H, compared to 1,831 kbp in the GBS marker set. However, the GBS marker set had more supporting markers and identified a second locus at the bottom of chromosome 6H. This locus was also above the threshold *p*-value in the 50K dataset, but below the adjusted FDR. This region along with two others on the top and bottom of 2H in both datasets and top of 5H in the 50K dataset may be worthy of further exploration as modifiers of the naked phenotype.

Third, we took the same approach to explore growth habit (flowering dependent upon a period of vernalization) using both marker types and a categorical classification of winter versus spring growth type. We observed a single well-supported (i.e., multiple significant SNPS) association on chromosome 5H [−log_10_(*p*)∼8.0] in the 50K dataset some 29 kbp from the vernalization gene *HvVRN1* (von Zitzewitz et al., 2005). In the GBS dataset, we found 29 more significant but less well-supported single SNPs broadly distributed across the genome with −log10(*p*) values of > 8 to < 20. Surprisingly none convincingly co-located with the *HvVRN1* 50K peak, with the closest of 8 markers distributed across 5H chromosome with −log10(*p*) > 8 some 50 Mbp from the *VRNH1* locus. Based on this small dataset we conclude that both platforms, in general terms, perform equally well with any differences being trait-specific (Table 2). Combining both datasets for GWAS may be expected to enhance the likelihood of detecting trait-specific significant and real associations.

**TABLE 2:** Genetic resolution of GWAS for row-type, growth habit, and hull adherence.

Traits	Associate SNP	Chr	Position	Technology	Gene	Minimum distance in bp
Row- type	JHI-Hv50k-2016-107445	2H	651,372,755	50K	VRS1	657,881
	2:651372029	2H	651,372,029	GBS		658,607
	JHI-Hv50k-2016-231001	4H	17,377,068	50K	INT-C	223,056
	4:17598761	4H	17,598,761	GBS		1,363
Growth habit	JHI-Hv50k-2016-335893	5H	598,787,735	50K	VrnH1	339,089
	5:648520473	5H	648,520,473	GBS		49,391,201
Grain Hull	JHI-Hv50k-2016-491472	7H	546,632,335	50K	NUD	44,963
	7:548419008	7H	548,419,008	GBS		1,831,636

### Discriminant Analysis of Principal Components (DAPCs)

To explore genetic drivers of the observed population structure we used DAPC (Jombart et al., 2010), a multivariate approach that identifies clusters of genetically related individuals based on genetic data and the contribution that individual factors (SNP alleles) make to the observed population subdivision. For each marker type the first two PC’s clearly distinguish three major groupings (GP1-GP3) (Supplementary Figure S3). Comparing the cluster assignment of individuals from DAPC highlighted 30 individuals that appeared to be discordant between the datasets and these were removed for further analysis. Of the concordant individuals, 459 fell into group1 (GP1), 246 into GP2 and 265 into GP3 (Supplementary Figure S3 and Supplementary Table S1). GP1 was comprised of mainly (92%) 6-row genotypes exhibiting both spring and winter growth habit and were classified as a combination of landraces and cultivars from across the geographical range (Supplementary Figure S4). GP2 was principally (78,5%) 2-row spring type landraces and cultivars from across the geographical range (Supplementary Figure S5) and GP3 (75,5%) six row spring types mainly from Asia (Supplementary Figure S6).

In addition to DAPC loading plots (Jombart et al., 2010, data not shown) we calculated Fst statistics independently for each marker between groups (GP1/GP2, GP1/GP3, and GP2/GP3) and displayed the resulting data as Manhattan plots in order to illustrate the drivers of differentiation (Supplementary Figure S7). Overall patterns generally appear similar from both datasets, with more support (markers) for differentiated regions in the 50K dataset most likely a result of the number of markers included in the analysis. Between GP1 and GP2 that contrast mainly for row-type we observed strong signals of differentiation around the row-type genes *HvVRS1* (2H) and *HvVRS5* (4H) and a complex region on chromosome 1H flanked by the same locus observed in the GWAS analysis for row-type (Figures 5A,B). A signal was also observed near the photo-period response gene *HvPPDH2* (Turner et al., 2005). For GP1 and GP3 that contrast mainly by geographical origin, the Fst revealed a complex set of highly significant signals largely unrelated to known major genes, with the possible exception of *HvVRNH1* on chromosome 5H and *HvCEN* on 2H that may stem from the mixed growth habits of the individuals in GP1. GP2 and GP3 differ largely according to row-type and geographical origin and once again revealed complex signals, including those close to *HvVRS1*, *HvVRS5*, *HvVRNH1*, and *HvCEN* that may be expected to differentiate these groups. In all comparisons there was very strong support for differentiation between groups at a tractable number of highly specific genomic regions that, based on our regional gene content analysis, do not contain well-known or characterized candidate genes which are ripe for further investigation.

### Relative Costs

A widely held opinion in the plant research community is that GBS is considerably cheaper than SNP-arrays. We were therefore interested to compare the relative costs of each technology when applied to exactly the same set of genetic materials. In our analyses (conducted in the United Kingdom) the cost of genotyping a single accession with the barley 50K SNP chip through a commercial vendor is £40 per sample (January 2019). On the basis of the experiment reported here this returned approximately 37,000 high quality markers, the vast majority with a MAF > 5% and therefore suitable for most common types of genetic analyses. *Pst*I/*Msp*I GBS can also be provided as a commercial service at a current cost of £60.50 per genotype (January 2019) based on a 1,000-sample size. We presume that these cover all necessary operational costs including staff, supplies, the original purchase and annual maintenance of necessary instrumentation, and in the case of GBS a license for commercial use^3^, but excludes other than basic data analysis. At this moment in time we consider this a realistic comparison. As the number of informative bi-allelic SNPs with MAF > 5% observed in the current GBS study is approximately half of that revealed by the 50K platform (following Milner et al., 2019), we conclude that for applications requiring this MAF, the cost for each informative bi-allelic SNP is significantly lower for barley using the 50K SNP chip (ca. 1/3 of the cost). Note however that this cost comparison excludes the considerable investment required for the development and fabrication of a high-quality SNP-array and that all quoted costs are subject to fluctuations over time.

## Discussion

The application of DNA based genotypic analyses to efficiently study genetic diversity in populations of plants underpins contemporary genetic analyses from germplasm evaluation, high-resolution genetic mapping and genomic prediction. If a simple pre-requisite is for the genetic data generated to be robust, reproducible and long lived then it is important that we understand the comparative values and features of different genotyping approaches. Here, we set out to compare two popular methods for genotypic analysis of plants, *Pst*I/*Msp*I GBS and Illumina Infinium^TM^ SNP-array technology, seeking to understand how they performed on a common set of 1000 diverse barley genotypes. When we started this piece of work we were unaware of any similar comparison having been conducted at a comparable scale and reported in the scientific literature. The 1000 barley genotypes that we examined originated from the German Federal *ex situ* GenBank hosted at IPK Gatersleben and represented the breadth of diversity from across the range of the species. GenBanks are vitally important international germplasm resources and given the considerable long-term investment they represent, it is important that we understand whether their characterization using one molecular approach (in this case GBS) provides an accurate overall reflection of genetic diversity. A detailed description of the 1000 genotypes and how they were identified and chosen as a core set is given in Milner et al. (2019) and a description of the design of the 50K SNP-array is provided in Bayer et al. (2017).

Starting with collections of 39,733 and 37,327 high quality SNPs distributed across the barley genome for 50K SNP-array and GBS datasets respectively, a number of features immediately became apparent. Firstly, the overlap between the datasets was restricted to 464 common markers highlighting the different type of sequences accessed by each approach. While the 50K SNPs were derived from exome capture data and therefore focused on coding sequence variation, the GBS data represents a wider survey of diversity in low copy genomic regions that are associated with low levels of DNA methylation. Although the latter regions may include many genes, our data indicates that the same SNPs are rarely sampled. Much gene level variation therefore appears to be excluded from the comparison. Secondly, applying MAF cutoff thresholds of <1 or <5%, the percentage of markers falling below these thresholds was 1.2 and 7.6% for the 50K chip but reached 41.6 and 58.9% for GBS, a clear reflection of the high number of low frequency alleles detected by the latter approach. Thirdly, the overall physical distribution of the markers revealed a much higher frequency of 50K SNPs in the gene-rich telomeric ends of each of the chromosomes reflecting the biased design strategy for the 50K chip (Bayer et al., 2017). The GBS SNPs showed a similar bias toward the telomeric ends, but it was less extreme. Furthermore, the GBS markers revealed considerably less genetic diversity (π) than the 50K SNPs, most likely a direct consequence of the low MAF. In contrast, rare SNPs were underrepresented in the 50K data which may be an issue for certain applications, for example GWAS of relatively rare phenotypes. Fourthly, the overall correlation between the diversity matrices was reasonably high (*r* = 0.62, *p* = 0.01, 99 permutations, Mantel test) suggesting that the overall sampling of diversity is well-represented by both approaches. This conclusion was supported by our GWAS results that indicated that, in the majority of cases, both approaches are likely to detect markers closely associated to genes controlling major phenotypic traits. Despite the apparent differences, the general message is that both perform comparably well in the types of analyses that we conducted here.

Comparisons between molecular approaches for revealing polymorphism have been extensively performed across a number of species (e.g., Lubberstedt et al., 2000; Uptmoor et al., 2003) including barley (Russell et al., 1997), but have mainly focused on early generation molecular assays (e.g., AFLP and SSRs). Only recently Elbasyoni et al. (2018), compared SNP-arrays with GBS for estimating genomic kinship and population structure, and assessing genomic prediction accuracy in 282 hexaploid winter wheat genotypes. Interestingly, a similar number of SNPs to those used here were analyzed (20,089 GBS SNPs and 39,674 array SNPs with MAF > 0.05). In their study, a Mantel test comparison of genetic diversity matrices revealed a strong positive correlation (*r* = 0.77; *p*-value < 0.0001; 1000 permutations) between the two datasets and PCAs revealed a similar topography across the main PC’s. Comparing the performance of SNP-arrays and GBS for genomic selection revealed that GBS data with up to 50% missing values improved genomic prediction accuracies and estimated breeding values for four traits when compared to both SNP-arrays and, somewhat curiously, GBS SNPs with only 10% missing values. These observations led the authors to conclude that GBS (with more missing data) is comparable to or better than SNP-array data for both genetic diversity and genomic prediction applications. The authors also commented that while SNP-arrays are reliable, robust marker platforms with low missing values, they have relatively high costs. However, a realistic cost comparison between the two approaches was not provided to support this assertion. There could be several reasons for the discrepancies between these two studies, including the ascertainment of SNPs on each array (e.g., the barley array used a larger and wider ascertainment dataset than the wheat array) (Moragues et al., 2010), the polyploid nature of the wheat genome, and the distribution of SNPs across the recombination landscape of the respective genomes.

Cost comparisons are surprisingly difficult to conduct at an organizational level due to a number of factors, including the resources available, how the work is done, the cost of supplies, access to appropriate equipment (and what it cost to purchase and to maintain it annually), whether – for GBS – a license from Keygene has been purchased^4^, how staff time costs have been calculated and so on. We used quotations for genotyping 1000 samples from two commercial service providers, making the assumption that they had conducted detailed costings to cover all associated costs (many of which may be hidden in an academic setting). The work of the contracting lab is therefore exactly the same – plant growth, DNA isolation, sending samples to the service provider and receiving the data. For barley we found that the per genotype cost was roughly equivalent for each technology, but the cost per informative SNP (i.e., co-dominant, MAF > 5%, < 10% missing data) from the 50K array was significantly less than that obtained by GBS. However, we acknowledge that this cost comparison may not hold for all species, particularly for those with more complex genomes (e.g., wheat) or less well-developed SNP-array technology.

Missing data in low coverage GBS leads to both missing calls and under-called heterozygotes. These are the product of a number of factors, the major technical ones being depth of sequence coverage (related to genome size) and the level of multiplexing required to provide the cost efficiencies generally assumed for the GBS protocol. Missing data may reflect unsampled sequences or genuine biological diversity in the form of null alleles [presence/absence variants (PAVs)] and may therefore have hidden genetic value. These alternatives are difficult to distinguish. To capture value from missing data, fast and accurate allele imputation programs such as FILLIN and FSFHap (Swarts et al., 2014) have been both developed and proven highly successful in accurately predicting allele calls in sparse GBS, large sample-size datasets. Unsurprisingly, they ideally require high coverage (low missing data) information from representative haplotype panels or very large datasets (numbers of individuals) to ensure haplotype generation and imputation is accurate, particularly as it’s impossible to impute minor alleles correctly if they are not replicated anywhere else in the overall population. While this may be straightforward for very large sample sets in a single study, our impression is that making subsequent comparisons to much smaller datasets conducted in a different lab with different germplasm and different filtering criteria/software may not be completely straightforward. An advantage of the 50K chip lies in its robustness and the simple comparability of data generated in independent studies.

Missing data and bad calls are also observed on even the best SNP chips and can similarly be the result of technical or biological factors such as the presence of close sequence-related paralogs, sequence polymorphisms in the assay footprint outside of the queried SNP causing the assay to fail, borderline quality DNA or the presence of genuine PAVs. Runs of physically adjacent missing datapoints in an otherwise technically sound SNP chip assay or population-based quality filtering for SNaPs (Gabur et al., 2018) can provide strong support for genuine PAV interpretation which can then be included in genetic analyses. We identified 623 markers in the 50K dataset that recorded missing data in more than 10% of the individuals (data not shown) while all other markers exhibited robust calls throughout each individual genome. While these are good PAV candidates, further work is required to distinguish which of the above explanations is true. It would also be interesting to test whether their inclusion as rare variants in GWAS (i.e., as a second or third allele) improved the power to detect genetic components of complex traits in barley, as recently shown in oilseed rape (Gabur et al., 2018).

We used two approaches to explore the use of each marker dataset for identifying the location of genes underlying specific traits, GWAS and DAPC. Similar results were obtained for GWAS with both marker types, perhaps with the exception of growth habit using GBS data which generated several poorly supported but highly significant associations. We also explored DAPC because we had previously and successfully used this approach to investigate the discrimination between the genetically narrow spring and winter 2-row elite NW European genepools (Comadran et al., 2012). The results revealed signals associated with genes that would be expected to differentiate the genepools (Supplementary Figure S7). Perhaps more significantly, the analysis also highlighted a tractable number of highly significant signals for which no current causal gene has been identified. While these are ripe for further investigation, this is outside the scope of the current report.

The use of SNP-arrays and GBS has already started to give way to (low coverage) whole genome shotgun sequencing in small genome models and crops (Wang et al., 2018). We attribute this largely to the availability of high-quality reference (pan)genome sequences and the generation of an appropriate depth of highly accurate sequence coverage that is easily attainable at today’s sequencing costs. However, for many of our major crops, especially those with large genomes, and for many applications, we predict that genotyping methods that efficiently and relatively cheaply sample relevant diversity, like GBS and SNP chips, will remain methods of choice for routine genetic analyses. For the intermediate sized 5 Gbp barley genome, we suggest that sequencing costs would need to drop by around an order of magnitude before whole genome shotgun sequencing at an appropriate depth could be routinely used as a genotyping platform that is capable of robustly revealing homo- and hetero-zygous loci. While this is clearly on the horizon, such a transition will require a fundamental shift in the skills and infrastructure available throughout the community (both fundamental and applied) to efficiently deconvolute and interpret whole genome sequence data. In a well-resourced research context, we argue the shift to whole genome survey sequencing is almost certainly attainable now, especially for populations that are used repeatedly to address a wide range of different scientific questions (e.g., GWAS, MAGIC, or NAM populations used by the academic community). If correct, the types of questions we raised as motivation for the current study, may soon become redundant.

## Conclusion

Both GBS and SNP-arrays efficiently sample the diversity present in the domesticated barley genepool but access different regions of the genome and reveal different characteristics. Choice of technology should be carefully considered according to desired applications and objectives, along with group preferences, available skills and infrastructure. Current commercial cost comparisons question the widely held view that GBS is considerably cheaper than SNP-arrays, in barley at least.

## Author Contributions

RW, NS, JR, LR, and MMas conceived the study. BD, SM, PH, PS, and MMac designed and coordinated the experiments and conducted the data analysis. NS, MMas, and SM chose and provided the germplasm. MMac and PH performed the lab experiments. RW, BD, JR, NS, and MMas wrote the manuscript, while CH, DF, and PL critically reviewed it.

## Conflict of Interest Statement

The authors declare that the research was conducted in the absence of any commercial or financial relationships that could be construed as a potential conflict of interest.

## Footnotes

1 https://www.illumina.com/content/dam/illumina-marketing/documents/products/technotes/technote_iselect_design.pdf

2 http://seedsofdiscovery.org/

3 COREHUNTER3 http://www.corehunter.org

4 https://www.keygene.com/work-with-keygene/licensing/
