# Supplementary material for: A Comparison of Mainstream Genotyping Platforms for the Evaluation and Use of Barley Genetic Resources
Source: Front Plant Sci. 2019 Apr 26;10:544. doi: 10.3389/fpls.2019.00544 (PMC6499090; doi:10.3389/fpls.2019.00544)
Supplement: Supplementary file 1 [file Table_1.DOCX]

**Supplementary Data**

**Figure S1: Sliding window analysis of SNP density**

**Figure S2:** **GAPIT - QQ plots for 50k and GBS data**

**Figure S3: DAPC groups GP1, GP2 and GP3 based on separation in PC1 and PC2**

**Figure S4: Principle coordinates analysis of GP1 (459 individuals)**

**Figure S5: Principle coordinates analysis of GP2 (246 individuals)**

**Figure S6: Principle coordinates analysis of GP3 (265 individuals)**

**Figure S7: Drivers of differentiation between groups**

**Table S1: Number of individuals in different Groups**

**Figure S1**: **Sliding window analysis of SNP density** in genic and intergenic regions revealed by the Illumina 50k SNP-array (left panels) and GBS (right panels) data from Milner et al (2018) along barley chromosomes 1H-7H according to physical position and genomic context (Red – genic, Blue - intergenic).


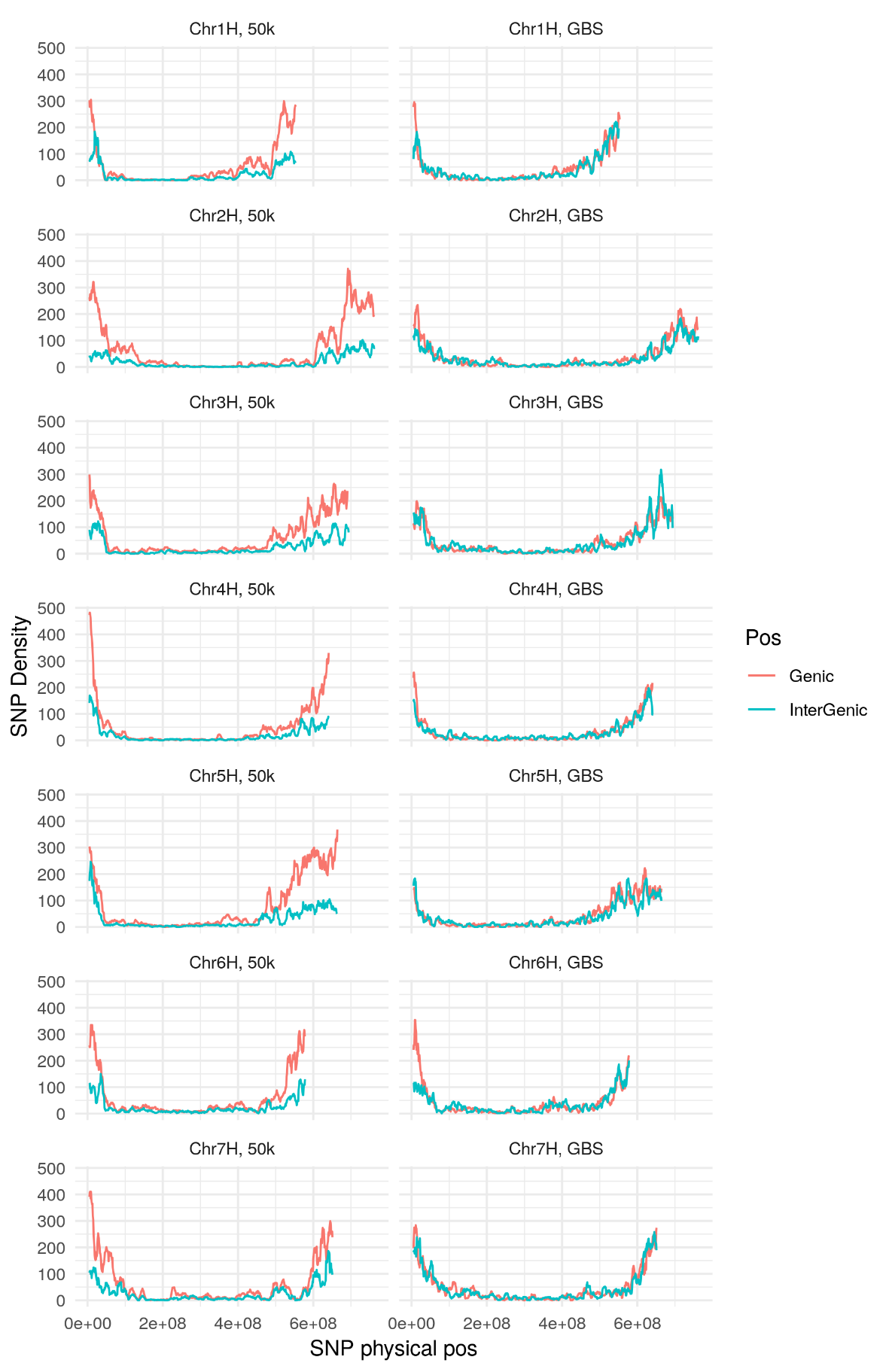


**Figure S2:** **GAPIT - QQ plots for 50k and GBS data** according to three life history traits


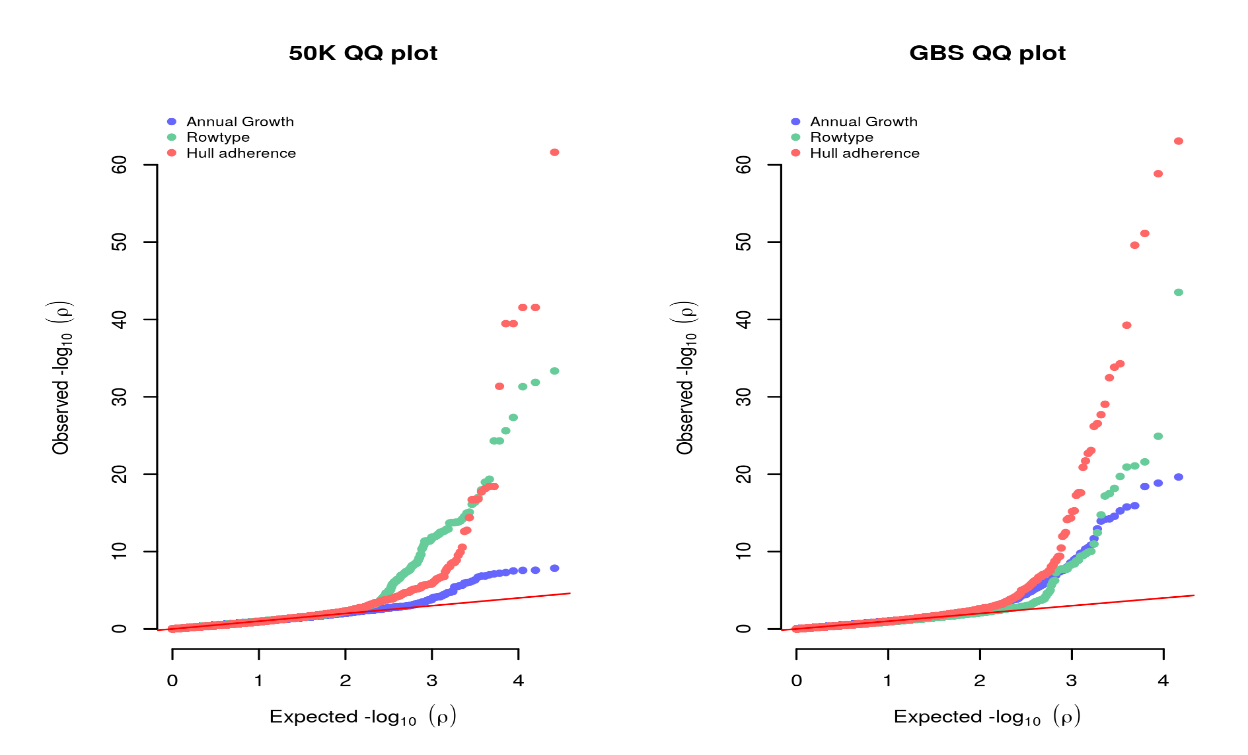


**Figure S3. DAPC groups GP1, GP2 and GP3 based on separation in PC1 and PC2.** GP1 (459 individuals) comprised a mixture of mainly 6-row genotypes (spring and winter) from all geographical locations, GP2 (246 individuals) was principally spring 2-row spring type landraces and cultivars from across the geographical range, and GP3 (265 individuals) six-row spring types mainly from Asia. 30 discordant individuals have been removed from this analysis (see main text)


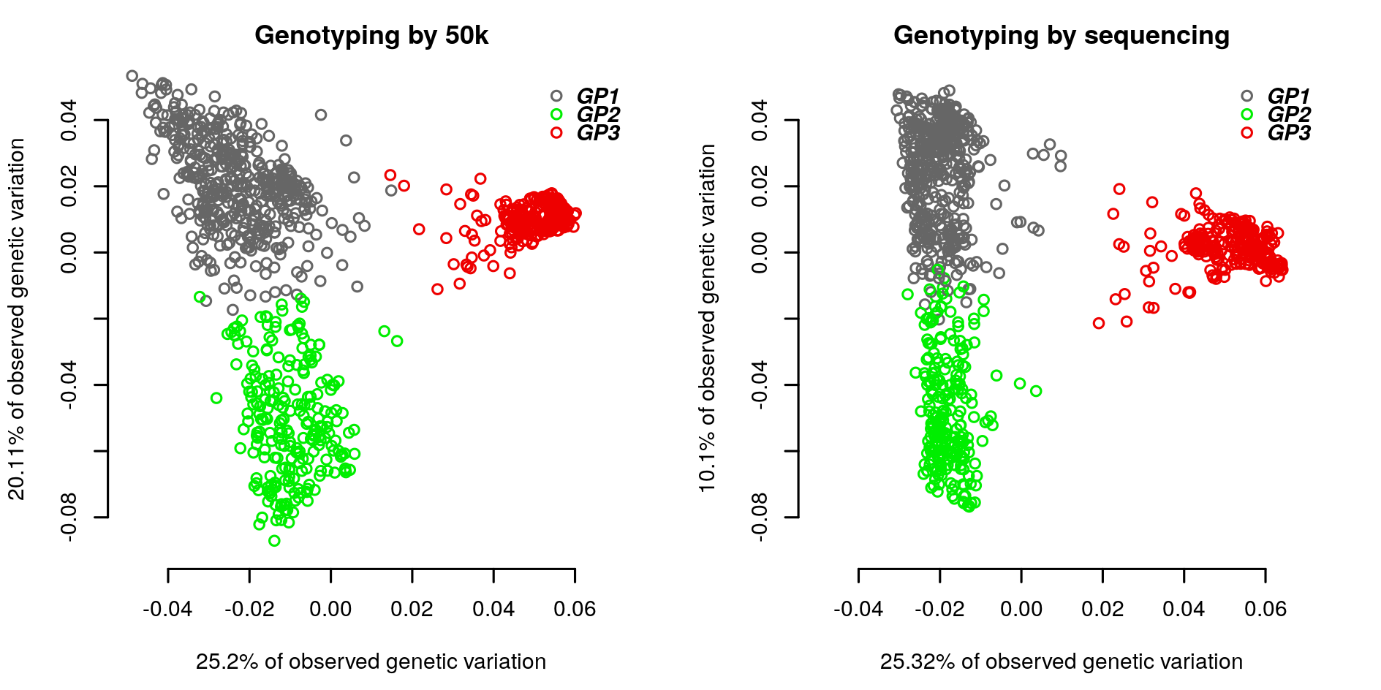


**Figure S4. Principle coordinates analysis of GP1** (459 individuals) based on 50K array (left panels) and GBS data (right panels). Partitioning according to geographical origin, growth habit, row type and landrace vs cultivar is shown (top to bottom).

**
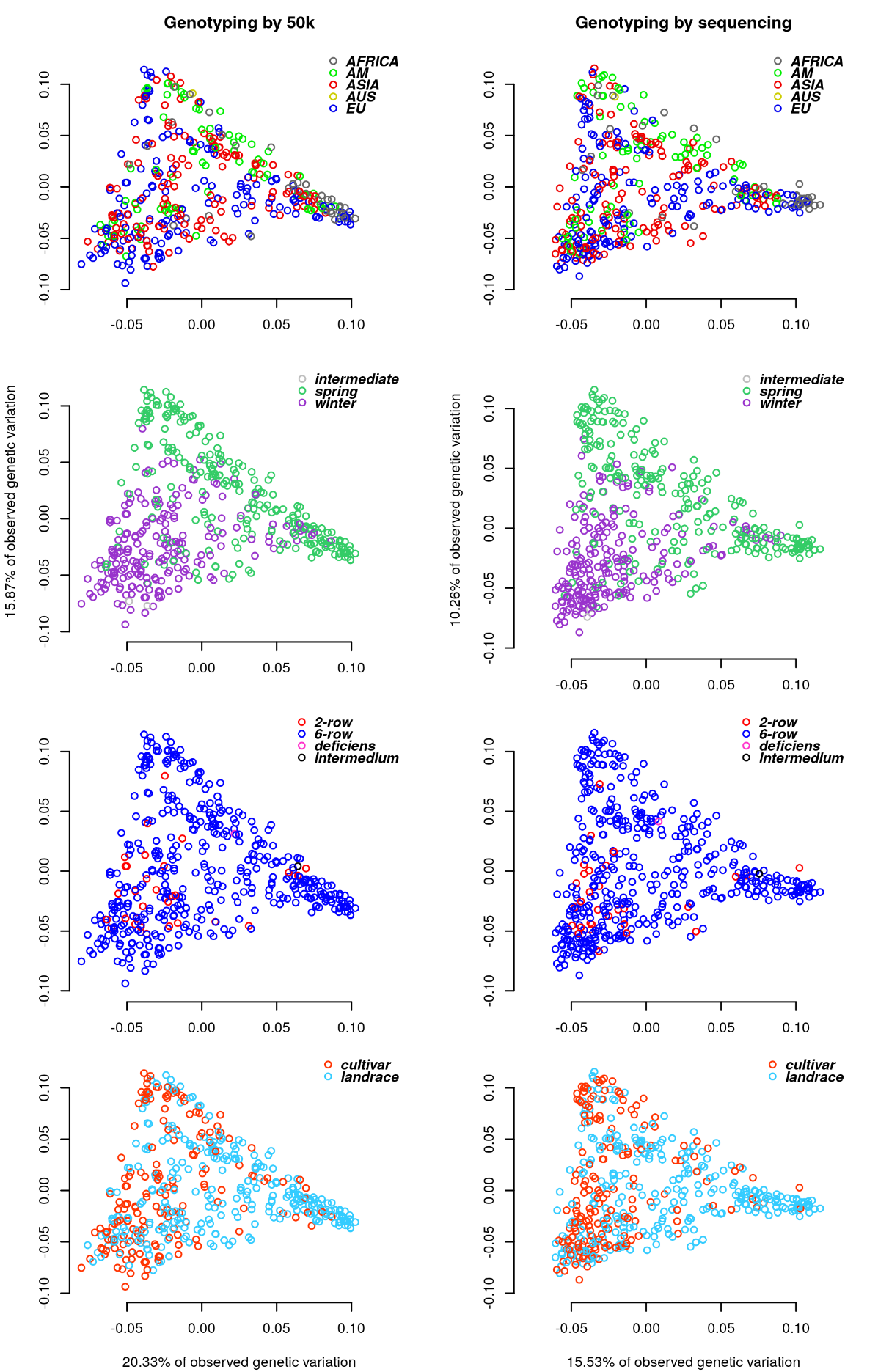
**

**Figure S5. Principle coordinates analysis of GP2** (246 individuals) based on 50K array (left panels) and GBS data (right panels). Partitioning according to geographical origin, growth habit, row type and landrace vs cultivar is shown (top to bottom).


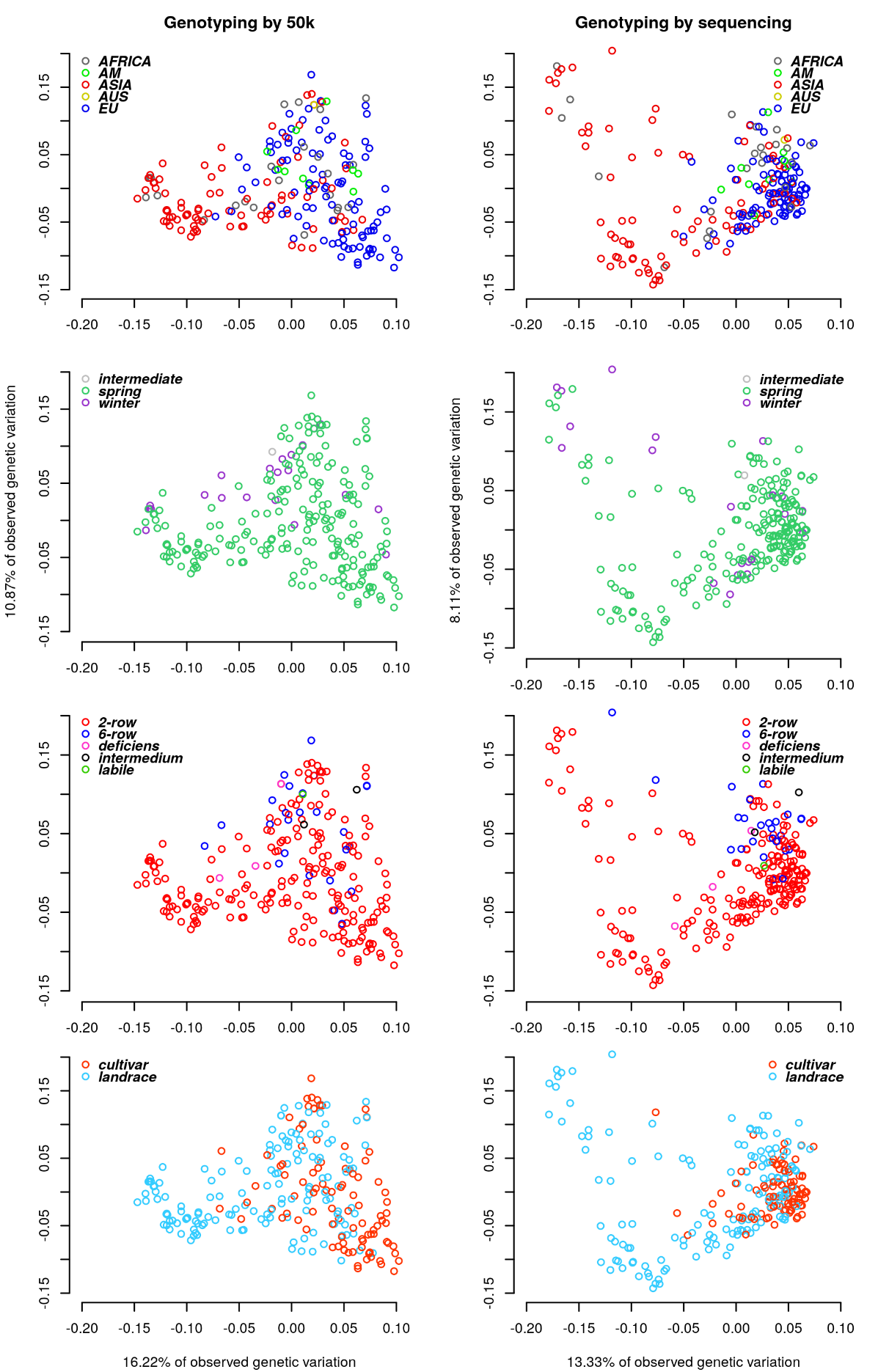


**Figure S6. Principle coordinates analysis of GP3** (265 individuals) based on 50K array (left panels) and GBS data (right panels). Partitioning according to geographical origin, growth habit, row type and landrace vs cultivar is shown (top to bottom).

**
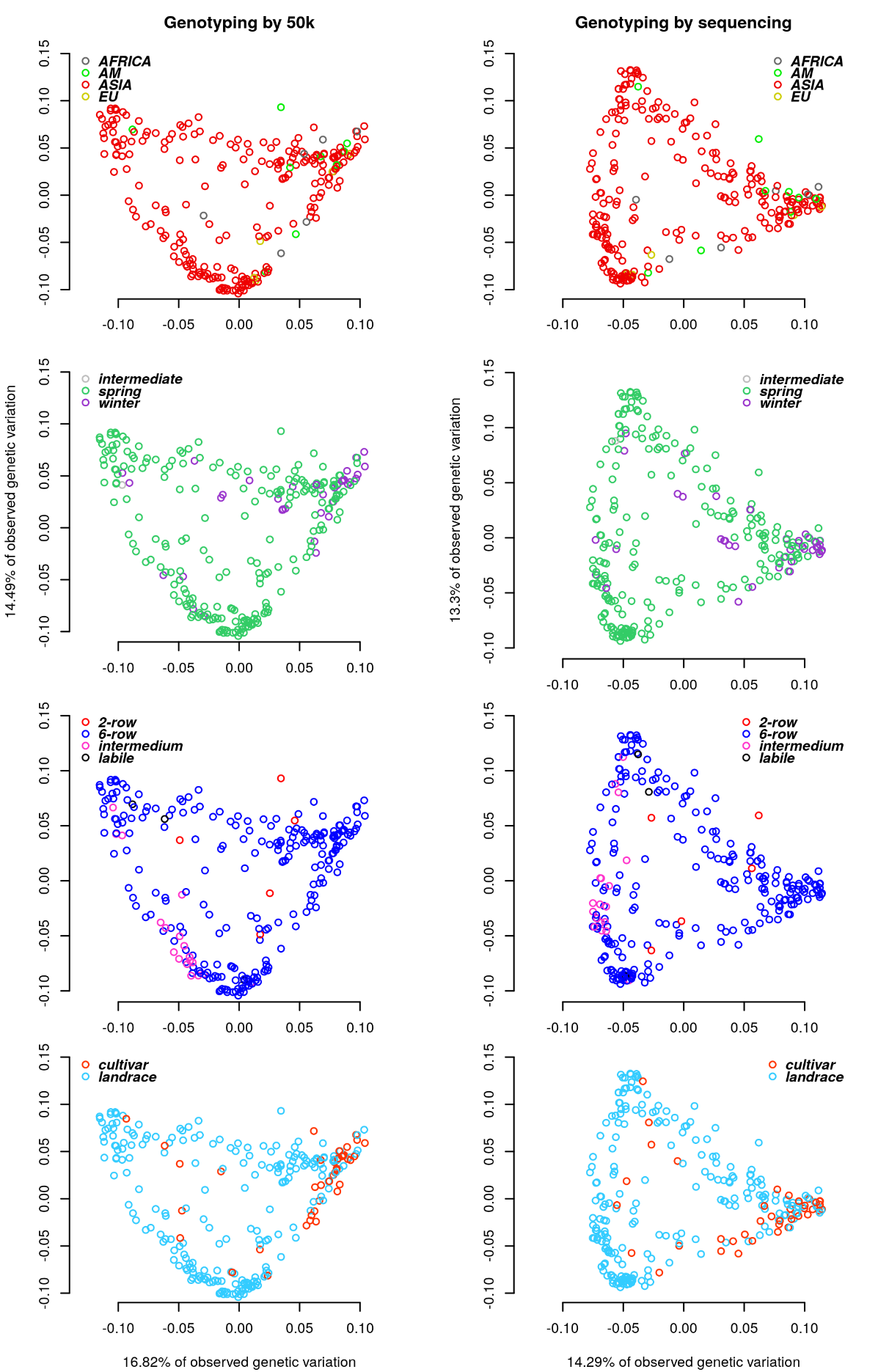
**

**Figure S7: Drivers of differentiation between groups a., b.** -log10(Fst) values for markers between GP1/GP2, **c., d.** between GP2/GP3 and **e., f.** between GP1/GP3 plotted as Manhattan plots. **a., c.** and **e**. are from the 50K array and **b., d.** and **f.** from the GBS dataset. Regions containing known genes related to specific traits are highlighted.


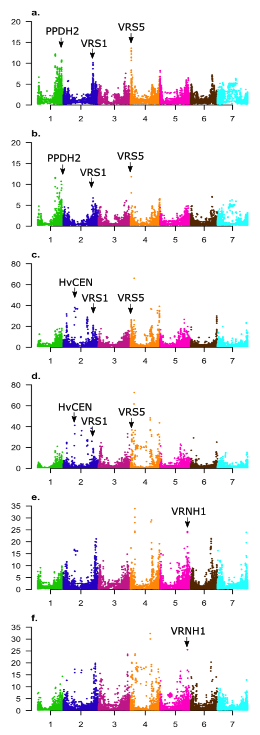


**Table S1: Number of individuals in different** **Groups**

| GP1 | 6-row | 2-row | Deficiens | Intermedium | NA | Total |
| --- | --- | --- | --- | --- | --- | --- |
| Winter | 190 | 22 | 0 | 0 | 0 | 212 |
| Spring | 230 | 8 | 1 | 1 | 2 | 242 |
| Intermedium | 2 | 0 | 0 | 0 | 0 | 2 |
| NA | 2 | 0 | 0 | 0 | 1 | 3 |
|  |  |  |  |  |  | 459 |

| GP2 | 6-row | 2-row | Labile | Deficiens | Intermedium | NA | Total |
| --- | --- | --- | --- | --- | --- | --- | --- |
| Winter | 6 | 18 | 0 | 0 | 0 | 0 | 24 |
| Spring | 19 | 193 | 1 | 3 | 2 | 1 | 219 |
| Intermedium | 1 | 0 | 0 | 0 | 0 | 0 | 1 |
| NA | 0 | 1 | 0 | 0 | 0 | 1 | 2 |
|  |  |  |  |  |  |  | 246 |

| GP3 | 6-row | 2-row | Intermedium | Labile | NA | Total |
| --- | --- | --- | --- | --- | --- | --- |
| Winter | 37 | 0 | 1 | 0 | 0 | 38 |
| Spring | 200 | 5 | 16 | 3 | 1 | 225 |
| Intermedium | 0 | 0 | 1 | 0 | 0 | 1 |
| NA | 0 | 0 | 0 | 0 | 1 | 1 |
|  |  |  |  |  |  | 265 |
